# Reducing computed tomography radiation dose in diagnosing pulmonary embolism

**DOI:** 10.12669/pjms.326.11347

**Published:** 2016

**Authors:** Tamader Y. AL-Rammah, Amani Alohaly, Kamal Albatsh

**Affiliations:** 1 Tamader Y. AL-Rammah, Department of Radiological Sciences, College of Applied Medical Sciences, King Saud University, Saudi Arabia; 2 Amani Alohaly, Department of Radiological Sciences, College of Applied Medical Sciences, King Saud University, Saudi Arabia; 3 Kamal Albatsh, King Khalid University Hospital, King Saud University, Saudi Arabia

**Keywords:** Pulmonary embolism, Computed tomography angiography, Effective dose, Dose length product, Computed tomography dose index

## Abstract

**Background and Objective:**

Computed tomography angiography plays a major role in the diagnosis of pulmonary embolism. Radiation dose associated with it is a major concern; therefore it is important to optimize protocols and techniques to ensure minimum radiation dose.

**Methods:**

The study compares two protocols i. Conventional Timing Bolus CT protocol and Delayed Timing Bolus protocol used to assist suspected pulmonary embolism patients.

**Results:**

A significant reduction in the average effective dose (39%) was noticed when using the delayed timing bolus protocol.

**Conclusion:**

Delayed timing bolus protocol has a good impact on radiation dose without affecting the value of the computed tomography angiography study.

## INTRODUCTION

Pulmonary embolism (PE) is a relatively common vascular disease.^1^ It occurs when clots in the systemic venous system break free and migrate to the pulmonary arteries, totally or partially occluding blood flow to the lung parenchyma.^2^

As the third most common cardiovascular cause of death after myocardial ischemia and stroke, PE has a common, potentially fatal condition associated with significant morbidity and mortality.^3^,^4^ PE has a high rate of mortality and accounts for 5% to 10% of all in hospital deaths.^5^-^7^ It is highly fetal and, in 22% of cases, it is not diagnosed before causing death.^5^,^8^,^9^

The non-specific signs and symptoms of PE, such as chest pain or shortness of breath which can be found in other diseases of the lungs, pleura, heart and gastrointestinal tract, making the diagnosis very challenging. Prompt and accurate diagnosis of P.E has been shown to greatly influence patient outcome, therefore, it is important to quickly and accurately diagnosis P.E.^10^

P.E. diagnosis relies on radiological imaging, the high spatial and temporal resolution of multidetector CT (MDCT) has allowed CT pulmonary angiography (CTPA) to supplant perfusion scintigraphy and catheter angiography.^4^ CTA has become a major diagnostic imaging procedure in patients suspected of PE.^11^ The high sensitivity and specificity, as reported by Henzier et al, where the largest study to investigate the use of CTPA in the diagnosis of P.E. has shown a sensitivity of 83% and specificity of 96%. Cost-effectiveness and 24 hour availability at most institutions have resulted in the preferential use of CT Pulmonary Angiography CTPA.^4^ Recent improvements in CT technology have shortened acquisition times to less than two seconds, providing relatively motion free images in patients who are short of breath, which is a common situation in patients suspected of PE, this resulted in less scanning repeats. CTPA provides direct visualization of the emboli as well as additional information relating to alternative diagnosis. However, optimal arterial opacification with contrast media is essential, but the availability of CT fast scan times allowed the visualization of the pulmonary vasculature in its peak contrast enhancement.

In recent years, physician and public awareness of radiation related cancer risk has increased dramatically.^4^ One study estimates that approximately 29.000 future cancers could be related to CT scan use in the USA in 2007 alone.^12^,^13^

Radiation risk becomes especially important in patient with non-life-threatening PE, and in young individuals, particularly females,^14^,^15^ as they are more sensitive to radiation exposure due to the increase amount of breast tissue in the radiation field. Radiation dose to the breast in chest CT has been calculated and directly measured, with a wide variation in reported average values, ranging from 10 to 70 mGy. This compares with an effective radiation dose equivalent of (0.6–2.5 mGy) for two view chest radiography and an average glandular breast dose of (3 mGy) for standard two-view screening mammography.^16^

The risk-to-benefit of CTPA with appropriate clinical indication nevertheless strongly favors use of the examination, even in women.^4^ CT examination protocols and techniques should be optimized to limit the radiation associated with the scan.^12^,^17^ Reduction in effective dose per examination will lead to an overall reduction in population dose.^2^,^18^

The purpose of this study was to compare radiation dose associated with two CTPE protocols used in our department. The first protocol was used in 2014 which is the conventional timing bolus CT protocol and the second is the delayed timing bolus protocol which was implemented in our department starting from 2015.

## METHODS

This study was performed at our radiology department and data was collected before and after changing the standard scanning protocol for the evaluation of P.E. The study was performed with institutional ethics committee review board approval, and the requirement for informed consent was waived.

### Patient selection

One hundred twenty consecutive patients who were referred to CT for pulmonary CT angiography for suspected P.E were blindly selected (60 patients underwent the old CTPA protocol in 2014 and other 60 patients who underwent the new CTPA protocol in 2015). Inpatient, outpatient, and emergency patient were all considered for this study.

### CTA protocol

**Fig.1:**
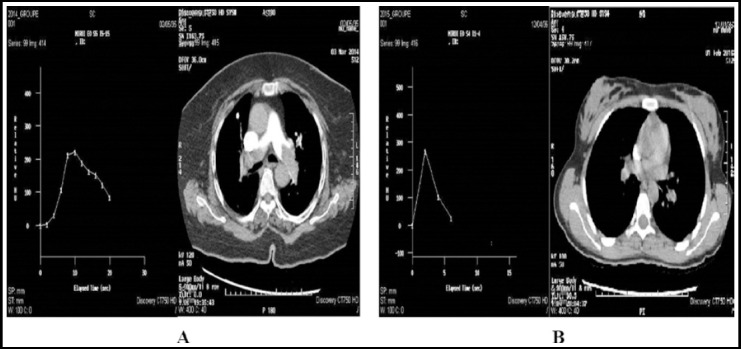
Peak enhancement of the a) timing bolus and b) delay timing bolus, the conventional technique need more time to reach the peak enhancement than the delay timing bolus.

All patients were scanned using 64-detector row (Light speed VCT GE Healthcare). Scan parameters were as follows: matrix 512X512, tube voltage 80-120, pitch 1,375 rotation time 0.6s. All imaging was acquired in a single breath hold in a caudocranial direction, started from the posterior costophrenic angles and ends at the lung apex. Patient received 40-100 ml iodinated contrast medium with 350 mgI/ml concentration (ominpaque350, GE Healthcare or xenetix350, Guerbet) with an injection rate of 5ml/sec. In 2014 the image delay was determined by automatic bolus tracking and selecting the best image time with the max contrast filing in the pulmonary artery. On the other hand the 2015 protocol has added waiting for five seconds before starting the scan for bolus tracking, as the contrast medium could not have reached the pulmonary artery in the first 5 sec and scanning in this time will be an addition of non useful radiation to the patient (Fig.1). Contrast medium volume was calculated to be equal to the product of the scan time delay and the flow rate. Adaptive Statistical Iterative Reconstruction (ASIR) algorithm was used; the image acquisition was modified by 30%.

### Scan assessment

Two experienced and blinded radiologists evaluated the images and identified patients with PE and all other findings; there was 100% interobserver agreement. DLP and CTDI volume were the parameter used to evaluate the change in the radiation dose to patients. Effective dose was estimated by multiplying the DLP by a coefficient of 0.014.^12^,^19^

## RESULTS

In this study we have included 120 suspected P.E patients all were referred to CT department to roll out possible P.E, timing bolus group were 60 and the delayed timing bolus group were also 60.

The mean age of the timing bolus technique group was 48.7years (SD ± 16.79, range 24-81), where 31 (51.7%) were female and 29 (48.3%) were male and the mean age of the delay timing bolus technique group was 53.1 years (SD ± 18.41, range 24-86), where 35 (58.3%) female and 25(41.7%) were male. A total of 26.7% of studies were positive for P.E in the timing bolus technique group, and 18.3% of studies were positive for P.E in the delay timing bolus technique group. The average CTDIvol for the timing bolus technique group was significantly higher than for the delay timing bolus technique group. The average DLP were significantly higher for the timing bolus group in comparison to the delay timing bolus group at p ≤ 0.001. The average effective dose was significantly higher in the first group 5.4mSv (SD 1.2, range 2.12-9.48) compared to the second group 3.3mSv (SD 1.2, range 1.8 – 8.44) (Table-I). The average effective dose for the delay timing bolus group was 39% less than that for the timing bolus group.

**Table-I:** Demographics and result compression.

	Timing Bolus	Delayed Timing Bolus	p value
Mean age	48.77 (± 16.79)	53.10(± 18.41)	0.160
Positive P.E (%)	16 (26.7)	11 (18.3)	0.120
CTDIvol (mGy)	73.84(±31.19)	46.33(±18.57)	≤0.001
DLP (mGy cm)	387.19(±87.46)	242.49(±90.89)	≤0.001
Effective dose (mSv)	5.4(±1.2)	3.3(±1.2)	≤0.001

## DISCUSSION

The risk of unnecessary radiation exposure has become an important issue, especially among young women, who may be exposed to substantial level of breast radiation or fetal radiation during pregnancy.^15^,^20^-^22^ For this reason there is an urgent need for the reduction of CT radiation doses.^20^

The changed in the protocol of pulmonary CT angiography for suspected P.E to delay timing bolus protocol in 2015 significantly reduced the effective radiation dose. We demonstrated a decrease in average effective dose of 39% in patients undergo the delay timing bolus protocol versus a conventional timing bolus CT protocol.

Comparatively, others have reported that using ASIR enabled the reduction of radiation dose in CT lungs, with preserved signal, noise, and study interpretability, in a large multicenter cohort. ASIR was associated with a 27% reduction in radiation dose compared with FBP represents a new technique to reduce radiation dose in coronary CTA studies.^12^,^23^ Another study reported that tube current, and thus radiation dose, could be reduced by 40% or 80% from ASIR or MBIR, respectively, compared with conventional FBP during CT lung cancer screening.^24^ ASIR technique was also associated with a greater than 57% mean dose reduction, without significantly impacting diagnostic image quality in pediatric chest CT examinations. Also the use of ASIR altered both the qualitative and quantitative assessment of smoking-related lung disease.^25^

Z-axis modulation appears to provide acceptable image noise and diagnostic acceptability with substantial tube current–time has been shown to reduce radiation dose by 18-26% at noise indexes of 10.0 (18%) and 12.5 (26%) HU compared with the fixed tube current technique.^12^ It has been reported that reducing the z-axis of CTA for P.E decreases the average effective dose by 49% that is higher than our study, however there are downsides to this approach. For instant, the potential for missing a small embolus that may occurs in the pulmonary arteries above the aortic arch and/or below the heart. Other potential concern is that significant, clinically important finding would be missed in the area above the aortic arch or below the heart.^12^ In our study we were able to decrease the effective dose without losing any important findings that may affect the diagnoses. In the timing bolus group 16 P.E. cases were detected and 33 other findings, and in the delayed timing bolus group 11 P.E. cases and 70 other findings (Table-II).

**Table-II:** Other findings in both protocols used.

Other finding	Timing Bolus	Delayed Timing Bolus
Consolidations	3	5
Atelectasis changes.	5	11
Pleural effusion	5	13
Opacity	3	9
Hepatic metastases	1	3
Lung metastases	1	4
Cardiomegaly	2	2
Cardiac dysfunction	1	0
Pulmonary hypertension	1	2
Hiatus hernia	1	0
Pneumonia	5	1
Pulmonary nodules	0	6
Infection	3	6
Pulmonary infarction	1	2
Pulmonary edema	1	0
Bone metastasis	0	2
Breast Lesions	0	1
Coronary arterial calcification	0	2
Pneumothorax	0	1

**Fig.2:**
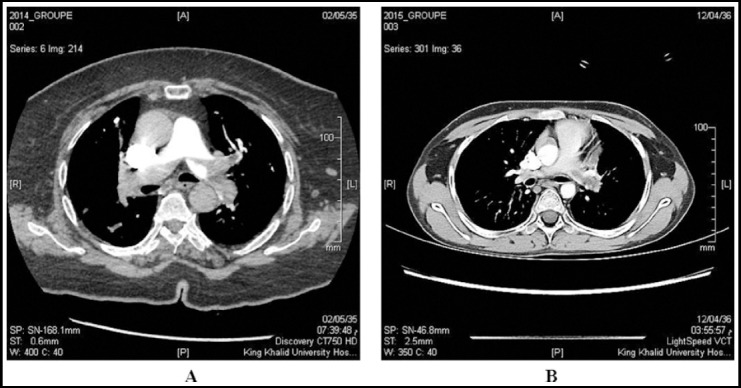
Image quality of a) timing bolus and b) delay timing bolus.

Additionally, in our study the decrease in the effective dose did not affect the image quality since there was no change in any of the scanning parameter (Fig.2). The decrease of the effective dose is only caused by the five seconds delay of the exposure after the injection of the C.M instead of starting the scanning immediately. This 5 sec is the time require for C.M to move from the auto injector to pulmonary artery, which is non-useful imaging.

## CONCLUSION

Delaying scan time for five seconds after contrast medium administration in CTPA, demonstrated a reduction in the CT radiation dose. This is a practical approach for achieving 39% decrease in effective dose without effecting image quality or missing any other important finding.
